# Spatiotemporal transcriptomics for tumour microenvironment remodelling in cancer progression and therapy

**DOI:** 10.1002/ctm2.70858

**Published:** 2026-10-11

**Authors:** Ying Kang, Qinjing Wang, Siqi Xu, Jiming Guo, Yue Ming, Weixing Chen, Min Wang, Zhijie Huang, Yisheng Huang, Bo Jia

**Affiliations:** ^1^ Stomatological Hospital, School of Stomatology Southern Medical University Guangzhou China; ^2^ School of Materials Science and Engineering East China University of Science and Technology Shanghai China

**Keywords:** precision treatment, spatial heterogeneity, spatiotemporal transcriptomics, time‐series analysis, tumour microenvironment

## Abstract

**Background:**

Spatiotemporal transcriptomics (STT) integrates spatially resolved gene expression with temporal information derived from staged samples, paired treatment specimens, or computational inference, providing a framework for reconstructing tissue‐level dynamics. In the tumour microenvironment (TME), STT extends static molecular and cellular maps by revealing how cellular neighbourhoods and functional niches are reorganised during tumour evolution.

**Main text:**

This review examines how STT characterises TME remodelling during malignant transformation, invasion, metastasis, ageing, and treatment response. We discuss strategies for resolving temporal dynamics, including stage‐stratified sampling, paired or serial specimens, pseudotime and trajectory inference, and complementary intravital imaging. We further consider how spatiotemporal analyses can connect changes in cellular states, spatial organisation, and intercellular interactions with clinically relevant tumour phenotypes.

**Conclusion:**

By linking the spatial organisation of the TME with stage‐ and treatment‐associated molecular changes, STT may improve the prioritisation of candidate targets, treatment stratification, and the development of precision oncology strategies.

## Highlights

- STT reconstructs tissue dynamics by integrating spatial profiles with temporal information inferred from discrete samples.
- It extends static snapshots to multi-stage analyses of tumor microenvironment heterogeneity and plasticity.
- STT complements intravital imaging by providing broader molecular coverage but lower temporal continuity.
- STT strategies clarify therapeutic resistance and support biomarker discovery, patient stratification, and precision oncology.

## Graphical abstract

This review summarises how spatial transcriptomics technologies reveal tumour microenvironment heterogeneity and support the inference of dynamic remodelling during cancer progression.

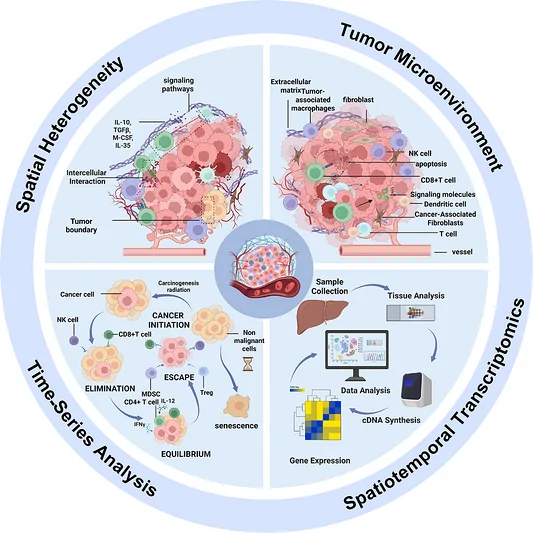

## 1 INTRODUCTION

Tumour microenvironment (TME) is a co‐evolving ecosystem whose spatial organisation and temporal plasticity are closely associated with tumour progression and therapeutic response.^1^ Rather than being a static collection of cells, the TME comprises multiple functional spatial niches, such as hypoxic, perivascular and pre‐metastatic niches (PMNs). Through their distinct cellular compositions, molecular signals and local environmental conditions, these niches shape tumour cell states, immune activity, metabolic adaptation and clonal evolution. As disease progresses and therapeutic interventions are applied, these spatial niches undergo continuous remodelling and may gradually develop immunosuppressive features that promote therapeutic resistance.^2^ Understanding tumour progression therefore requires not only identifying the cellular and non‐cellular components of the TME, but also determining how they are spatially organised and how their interactions change over time.

Bulk transcriptomic analysis averages molecular signals across tissue regions, whereas dissociation‐based single‐cell RNA sequencing separates cells from their native spatial context. Conventional spatial transcriptomics partly addresses these limitations by mapping gene expression within intact tissue architecture, but most spatial datasets represent individual time points.^3^ Spatiotemporal transcriptomics (STT) extends this framework by integrating spatial transcriptomic profiles collected across disease stages, primary and metastatic lesions or treatment time points, together with computational approaches that infer temporally ordered changes.^4^ Because spatial transcriptomic sequencing is generally destructive, current STT studies usually do not continuously follow the same cells or tissue regions.^5^, ^6^ Instead, temporal relationships are reconstructed through comparisons of independent stage‐specific samples, paired pre‐ and post‐treatment specimens or trajectory‐based analyses.^7^ Longitudinal intravital imaging provides direct temporal observation of cellular behaviour, whereas STT offers broader molecular coverage within preserved tissue architecture.^8^ These approaches are therefore complementary.

By linking molecular states to spatial locations, STT can reveal changes in the abundance, distribution and functional states of tumour, immune, stromal and vascular populations during disease progression or treatment. It can also identify region‐specific signalling and metabolic programs associated with immune suppression, invasive growth, metastatic niche formation and therapeutic resistance. However, because many temporal relationships and cell–cell interactions are computationally inferred, these findings should be interpreted in the context of the sampling design, analytical methods and available experimental validation.^9^ We organise this review around spatial niches and disease‐ or treatment‐associated changes because the biological relevance of molecular programs is often critically influenced by their location, cellular context and timing. This approach allows us to connect specific biological questions with the analytical methods used to address them and to assess the strength of the supporting evidence.

Against this background, this review examines the application of STT to TME remodelling during cancer progression and treatment. We first describe the major cellular and non‐cellular components of the TME and their organisation into functional niches. We then summarise the basic principles, major platforms and analytical workflows of STT, with particular attention to the reconstruction of temporal relationships from multi‐stage or treatment‐related spatial datasets. Subsequent sections discuss spatial heterogeneity and temporal changes in immune, stromal, vascular and metabolic programs during tumour initiation, invasion, metastasis, aging and therapeutic resistance. We further consider the potential applications of STT in therapeutic target identification, immunotherapy optimisation and patient stratification, as well as the methodological limitations that affect the interpretation and translation of current findings. Unlike previous reviews that primarily summarise spatial transcriptomic platforms or their applications in oncology, this review focuses on incorporating temporal evidence into spatially resolved studies of TME remodelling. We define STT as an inferential framework and distinguish direct longitudinal observation from temporal relationships reconstructed using stage‐stratified samples, serial specimens or computational state ordering.

## 2 OVERVIEW OF TME

Tumour formation results from complex interactions among cancer cells, infiltrating immune cells, stromal cells, blood vessels, the extracellular matrix (ECM) and secreted factors, often under pathological conditions such as hypoxia. Together, these components constitute a dynamic and heterogeneous biological network known as the TME.^10^ Within the TME, various cell types secrete bioactive molecules through paracrine and autocrine signalling, shaping a pathological signalling network that promotes angiogenesis, immune suppression and matrix remodelling, thereby facilitating tumour progression.^11^ Notably, these molecular and cellular interactions vary substantially across different spatial regions of the tumour and at different stages of disease progression, reflecting pronounced spatiotemporal heterogeneity (Figure 1).

**FIGURE 1:**
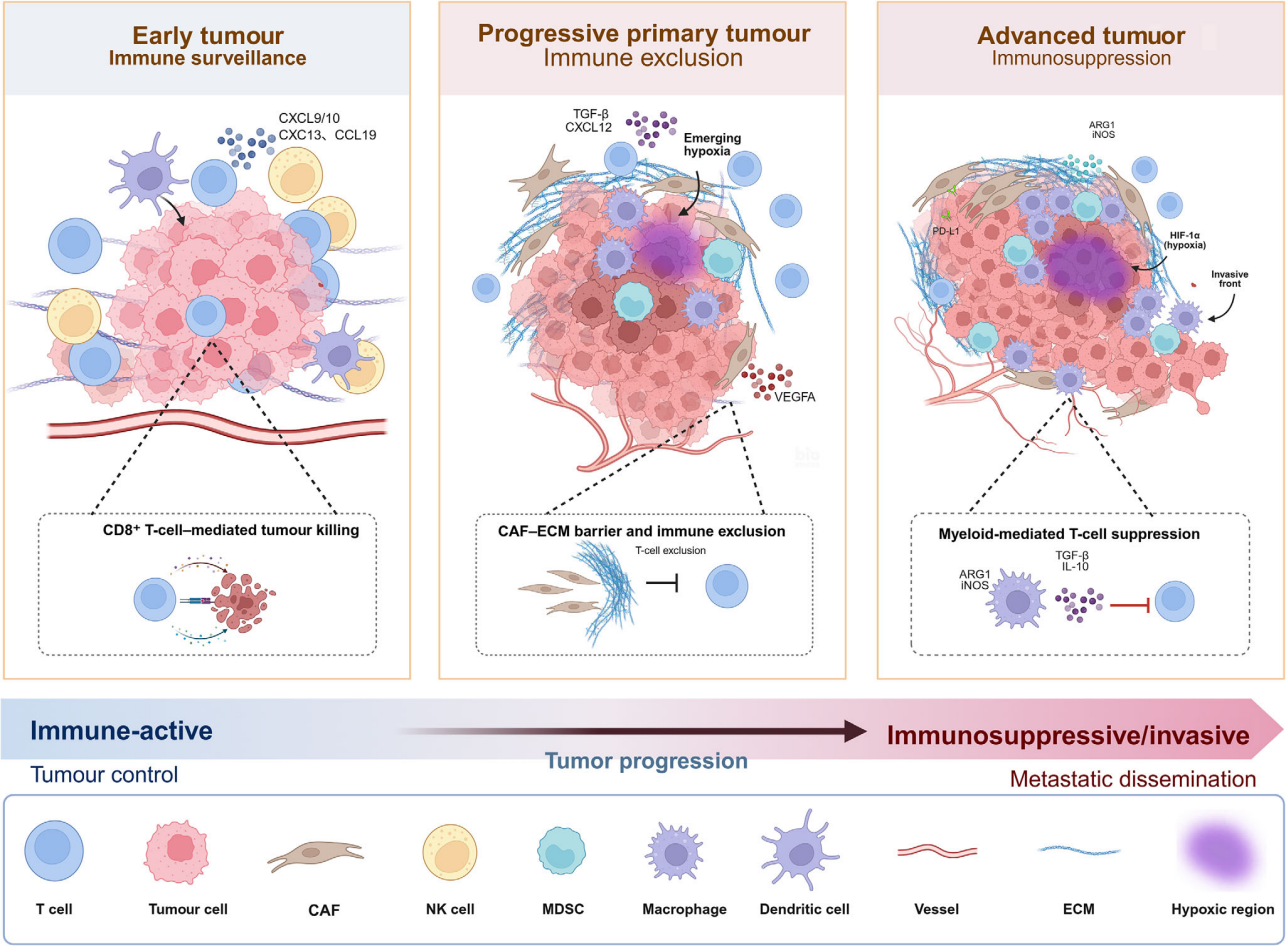
Composition and temporal evolution of the tumour microenvironment. Tumour progression is associated with a shift from immune surveillance in early tumours to immune exclusion in progressive primary tumours and immunosuppression in advanced tumours. These changes involve cancer‐associated fibroblast (CAF)–extracellular matrix (ECM) barrier formation, hypoxia, vascular remodelling and myeloid‐mediated T‐cell suppression, together with signalling programs associated with invasion and metastasis.

### 2.1 Tumour cells

As a central component of the TME, tumour cells exhibit pronounced genetic and epigenetic heterogeneity and dynamically interact with the surrounding microenvironment throughout disease progression, thereby influencing tumour initiation, metastasis and therapeutic response.^12^ During this process, metastasis‐initiating cells acquire invasive capabilities through epithelial–mesenchymal transition, enter the circulation and colonise distant organs. After colonisation, disseminated tumour cells may enter a dormant state and later become reactivated following the establishment of an immunosuppressive microenvironment. This dynamic process depends on the continual remodelling of local microenvironmental signals and is characterised by marked spatial and temporal context dependence.^13^

### 2.2 Immune cells

As tumours progress from early lesions to invasive and metastatic stages, the TME gradually shifts from a state of immune surveillance to one of immune suppression. This transition is characterised by dynamic remodelling across distinct spatial regions and disease stages.

In early tumours or at the invasive margin, natural killer (NK) cells and effector T cells remain relatively active and can mediate antitumour responses.^14^ However, as tumour progression continues and hypoxic regions expand, these cells gradually become functionally exhausted and spatially restricted within the tumour core, resulting in diminished antitumour activity. Meanwhile, tumour cells reinforce the immunosuppressive landscape over time by upregulating immune checkpoint molecules and promoting the infiltration of regulatory T cells.^15^

NK cells mediate the recognition and direct elimination of early malignant cells through multiple cytotoxic mechanisms, while also triggering apoptosis in tumour cells.^16^ Within the TME, NK cells engage in complex interactions with diverse cellular components and signalling molecules, which suppress their cytotoxic activity and restrict their infiltration into tumour tissues.^14^ Owing to the presence of multiple immunosuppressive factors in the TME, the therapeutic efficacy of NK cells in solid tumours remains limited. These constraints hinder the full realisation of their antitumour potential and thereby compromise tumour control.^17^

Under conditions of persistent inflammation and metabolic stress, dendritic cells (DCs) progressively remain in an immature state, resulting in reduced antigen‐presenting capacity and consequently impaired activation of effector T cells.^18^ Tumour‐associated macrophages (TAMs), by contrast, exhibit stage‐dependent phenotypic transitions. They are predominantly pro‐inflammatory M1‐like cells at early stages, but gradually shift towards an immunosuppressive M2‐like phenotype under chronic inflammatory and hypoxic stress, with enrichment at the invasive front and in late‐stage lesions.^19^

Myeloid‐derived suppressor cells (MDSCs) are important immunosuppressive myeloid cells in the TME and increase gradually during tumour progression.^20^ Spatially, MDSCs are often enriched in hypoxic regions, invasive fronts, perivascular areas and PMNs. In these regions, they suppress cytotoxic T cells and NK cells through arginase‐1, inducible nitric oxide synthase, reactive oxygen species, PD‐L1 expression and immunosuppressive cytokines. MDSCs can also promote regulatory T‐cell expansion, angiogenesis, ECM remodelling and PMN formation.^21^ Through these effects, they contribute to immune evasion, tumour progression and therapeutic resistance.

Accordingly, differences in the spatial distribution and functional states of immune cells, together with their stage‐dependent transitions during disease progression, collectively constitute a continuously evolving immune ecosystem.

### 2.3 Extracellular matrix

During tumour progression, tumour cells continuously modulate non‐malignant cells within the TME, including resident stromal cells, through soluble factors and direct cell–cell contact. This regulation becomes progressively reinforced over the course of disease evolution and drives structural remodelling of the ECM, thereby creating conditions favourable for tumour growth and proliferation.^22^

For example, cancer‐associated fibroblasts (CAFs), a major stromal cell subset within the TME, influence tumour cell migration and invasion through ECM remodelling, while also shaping local immune responses by regulating the spatial distribution and functional states of T cells. As CAFs expand across distinct spatial regions of the tumour and as disease progresses, their functional states undergo dynamic changes, contributing to the coordinated evolution of stromal architecture and immune regulation.^23^

### 2.4 Vascular system

Within the TME, vascular remodelling supplies tumour cells with essential nutrients and growth factors, suppresses antitumour immune responses and promotes tumour growth, invasion and metastasis. During early tumour progression, the expression of pro‐angiogenic factors, such as vascular endothelial growth factor (VEGF), is upregulated in tumour tissue, resulting in increased vascular density to satisfy the metabolic demands of tumour cells.^24^

The lymphatic system plays a crucial role in maintaining fluid balance, removing metabolic waste and mediating immune surveillance. However, in tumour tissues, lymphatic vessels undergo abnormal remodelling and may be accompanied by abnormal dilation and rupture of lymphatic vessels. These changes impair lymphatic flow and drainage, hindering the effective clearance of tumour cells and their metabolic byproducts. Consequently, tumour cells enter the lymphatic system through lymphatic vessels, migrate to regional lymph nodes and establish metastatic foci, thereby promoting tumour dissemination.^25^, ^26^

### 2.5 Signalling molecules and metabolic products

Signalling molecules within the TME, including VEGF, tumour necrosis factor‐α, interferons, adhesion molecules and others, stimulate tumour cell proliferation, migration and invasion by modulating immune responses and intercellular adhesion.^27^ Immunosuppressive molecules such as PD‐L1, expressed by tumour cells and suppressive immune cells within the TME, inhibit antitumour immune activity and enable tumour tissues to evade immune surveillance.^28^

Hypoxia, a hallmark of most solid tumours, induces abnormal cellular metabolism within the hypoxic microenvironment, leading to the accumulation of intermediate metabolites such as lactic acid, ammonia and lipids. These metabolites influence tumour‐cell growth and metastasis by altering the acid–base balance, redox state and nutrient supply of the microenvironment.^29^, ^30^ For example, high concentrations of lactic acid acidify the TME, thereby promoting tumour‐cell invasion and metastasis. Metabolic abnormalities, including altered amino acid metabolism and enhanced nucleotide metabolism, also occur within the TME and affect tumour progression.^31^

Therefore, elucidating the dynamic changes in these molecular networks across spatial and temporal dimensions is essential for understanding the mechanisms of tumour progression and optimising precision therapeutic strategies.

### 2.6 Functional niches in the TME

With advances in spatial analysis technologies, the concept of functional niches has become increasingly important in tumour research. Rather than viewing the TME as a simple collection of individual cell types, this concept emphasises how different cellular and non‐cellular components are spatially organised into multicellular units with specific biological functions. Functional niches are multicellular regions with specific cellular organisation, communication networks and molecular features. Examples include hypoxic niches, perivascular niches, immune‐excluded niches, invasive‐front niches, CAF‐rich stromal niches, tertiary lymphoid structure (TLS)‐like immune hubs and PMNs. Each niche contains multiple interacting components and imposes distinct selective pressures on tumour and immune cells.^32^

Hypoxic niches often contain HIF‐driven tumour cells, suppressive macrophages, MDSCs, abnormal vasculature, lactate accumulation and ECM remodelling.^20^ Invasive‐front niches frequently include EMT‐like tumour cells, CAFs, TAMs, MDSCs and chemokine gradients that support tumour migration and local immune evasion.^33^ Immune‐excluded niches are characterised by cytotoxic lymphocytes retained at stromal borders, dense ECM barriers, suppressive myeloid cells and signalling axes such as TGF‐β or CXCL12–CXCR4. Perivascular niches bring endothelial cells, pericytes, immune cells and stromal cells into close spatial proximity, thereby regulating oxygen delivery, immune‐cell trafficking and therapeutic access.^32^

Together, these niches explain why the same TME components can produce different biological outcomes depending on their spatial arrangement and temporal remodelling. Tumour progression and drug resistance arise from spatially constrained interactions among cells, vessels, matrix, soluble mediators and metabolites. By preserving tissue architecture, spatial and STT approaches can define where these niches occur, how they communicate and how they change across disease stages or under therapeutic pressure. This framework provides a biological foundation for the later discussion of STT in tumour evolution, immune remodelling, metabolic adaptation and precision oncology.

## 3 STT TECHNOLOGIES AND APPLICATIONS

The spatial and temporal heterogeneity of the TME described above highlights the need for analytical approaches that preserve tissue architecture while reconstructing changes across disease stages and treatment conditions. STT is not a single experimental platform, but an analytical framework that extends spatial transcriptomics by incorporating temporal information derived from disease stages, treatment‐related sampling, molecular‐state ordering or complementary dynamic measurements. Conventional spatial transcriptomics generates molecular profiles from tissue sections while preserving their original spatial coordinates. STT further places these spatial profiles within a temporal or state‐transition context, allowing researchers to compare how cellular composition, transcriptional states and tissue organisation differ across biological conditions.

However, most current STT studies do not continuously track the same cells or tissue regions over time. Because spatial transcriptomic profiling is generally destructive, temporal relationships are commonly reconstructed from discrete tissue samples collected at different disease stages or treatment time points, or inferred computationally from transcriptional similarities among cellular states. STT therefore provides an inferential reconstruction of tissue‐state changes rather than direct real‐time observation. Understanding this distinction is essential for selecting appropriate experimental platforms, analytical strategies and biological interpretations.

### 3.1 Fundamental principles of STT

The experimental foundation of STT is derived from spatial transcriptomics, which localises and quantifies RNA molecules within intact tissue architecture.^34^ Spatial transcriptomic technologies can be broadly divided into sequencing‐based spatial transcriptomics (SST) and imaging‐based spatial transcriptomics (IST). SST combines spatial barcoding with high‐throughput sequencing, whereas IST uses fluorescence in situ hybridisation or in situ sequencing to detect RNA molecules directly within tissue sections.^35^

SST platforms, including Visium, Slide‐seq and Stereo‐seq, provide broad transcriptomic coverage across relatively large tissue areas, making them suitable for identifying spatial domains, comparing tissue regions and analysing multicellular organisation. Nevertheless, their effective cellular resolution depends not only on the nominal size of the capture unit but also on transcript‐capture efficiency, tissue quality, sequencing depth and downstream deconvolution or segmentation.^34^ For example, high‐density platforms such as Visium HD provide substantially improved spatial resolution, but accurate assignment of transcripts to individual cells still requires reliable image segmentation and cell‐type annotation.^36^

IST approaches can achieve single‐cell or subcellular localisation of individual RNA molecules and are particularly suitable for examining cellular boundaries, local neighbourhoods and intracellular transcript distribution. However, many imaging‐based platforms rely on predefined gene panels, and increasing the number of targeted genes can increase imaging time, data volume and analytical complexity. Thus, higher spatial resolution does not necessarily correspond to broader molecular coverage or greater detection sensitivity.^37^

Platform selection thus entails careful trade‐offs among spatial resolution, transcript detection sensitivity, gene coverage, tissue area, sample compatibility, throughput and cost.^38^ Nominal feature sizes vary widely across platforms. Conventional Visium^39^ uses spots approximately 55 µm in diameter, which often encompass multiple cells, whereas Slide‐seqV2^40^ employs approximately 10 µm features and Stereo‐seq provides submicrometre capture‐site spacing. As an illustrative example, Slide‐seqV2 achieves an RNA capture efficiency of approximately 50% of that obtained with scRNA‐seq while maintaining spatial resolution close to the cellular level.^41^ However, reducing capture unit size does not inherently improve effective sensitivity; smaller units yield fewer transcripts per spot, and spatial binning may be necessary to achieve adequate molecular depth. In contrast, imaging‐based platforms enable single‐cell or even subcellular localisation of targeted transcripts, yet their gene coverage and throughput remain limited by panel size and the requirement for sequential imaging cycles. Consequently, no currently available platform simultaneously delivers whole‐transcriptome coverage, subcellular resolution, high detection efficiency, large‐area tissue profiling and high sample throughput. Platform choice should therefore be driven primarily by the biological question at hand. Investigations of tissue remodelling across disease progression may emphasise broad tissue coverage and cross‐sample reproducibility, whereas studies of tumour–immune interfaces or subcellular signalling pathways may prioritise cellular or subcellular resolution. Key characteristics and limitations of representative platforms are summarised in Table 1.

**TABLE 1:** Commonly used spatial transcriptomics technologies and their characteristics.

Type	Platforms	Spatial resolution	Gene coverage	Characteristics	Refs.
SST	Visium	Multicellular level	Full transcriptome	Limited resolution; High throughput; Compatible with FFPE; Commercially mature.	^39^
	Slide‐seq	Near single‐cell level	Full transcriptome	High resolution; Specialised equipment; Lower detection sensitivity.	^42^
	Stereo‐seq	Subcellular level	Full transcriptome	Ultra‐high resolution; High throughput.	^43^
	GeoMx	Multicellular level	Targeted	Supports manual ROI selection; Compatible with FFPE; Low cost; Suitable for functional region analysis.	^44^
	FISSEQ	Subcellular level	Targeted	Unbiasedness; Lower detection sensitivity; Extended cycle.	^45^
	STARmap	Subcellular level	Targeted	Supports three‐dimensional (3D) spatial modelling; Thick tissue section analysis.	^46^
IST	MERFISH	Subcellular level	Targeted	High multiplexing capacity; High spatial resolution; Complex probe design.	^47^
	CosMx SMI	Subcellular level	Targeted	High spatial resolution; Compatible with FFPE; Suitable for targeted panels.	^1^
	Xenium	Subcellular level	Targeted	High sensitivity; High‐resolution imaging; Compatible with FFPE; Requires dedicated instrument.	^48^
	SeqFISH^+^	Subcellular level	Near full transcriptome	Ultra‐high gene coverage; Labour‐intensive; High computational burden; High spatial resolution.	^49^

STT also differs fundamentally from longitudinal intravital imaging. Intravital imaging can repeatedly observe the same tissue region and directly record processes such as cell migration, vascular remodelling and transient cell–cell contact, thereby providing substantially greater temporal continuity.^8^ However, its molecular multiplexing capacity and transcriptome‐wide coverage remain limited. By contrast, STT provides high‐dimensional molecular profiles but usually relies on destructive sampling and discontinuous observations. These approaches should therefore be regarded as complementary. Longitudinal imaging provides direct temporal continuity, whereas STT provides molecular depth and spatially resolved transcriptional information.

### 3.2 Temporal inference and analytical workflow

An STT workflow should distinguish pre‐processing of spatial data from inference of temporal relationships. After quality control, image registration, segmentation or spot deconvolution and cell‐state annotation, temporal evidence can be introduced through four main strategies, including ordered stage‐specific samples; paired or serial treatment specimens; state‐based trajectory or pseudotime inference; and kinetic approaches such as RNA velocity or metabolic labelling. These strategies are not interchangeable and support different levels of temporal inference.^3^, ^50^, ^51^, ^52^ Figure 2 presents the overall workflow from spatial measurement and temporal evidence to interpretation and validation, whereas Table 2 compares the evidence, supported inferences and principal limitations of each strategy.

**FIGURE 2:**
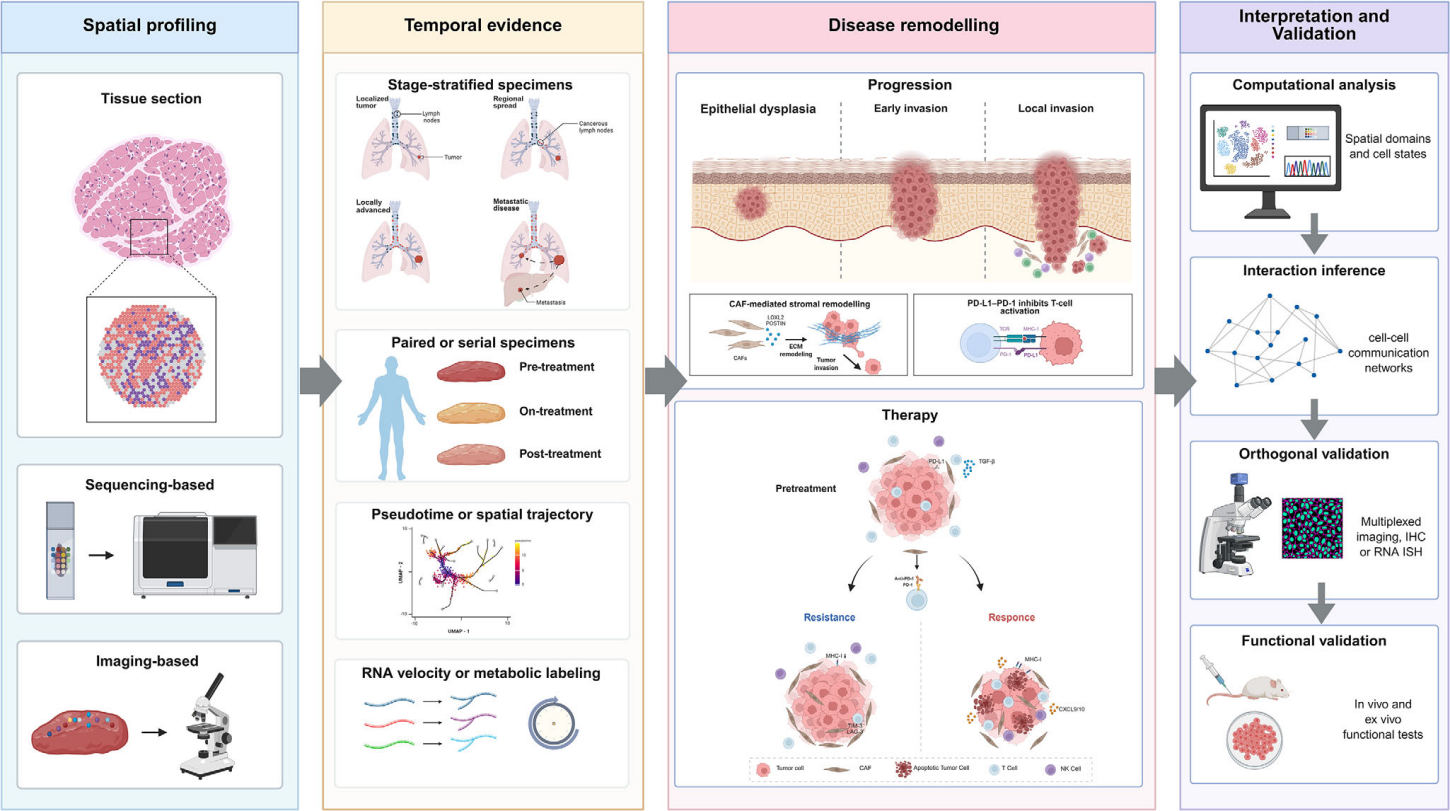
Workflow from spatial measurement to temporal interpretation and validation in spatiotemporal transcriptomics (STT). Following sequencing‐ or imaging‐based spatial measurement, temporal evidence is integrated from stage‐stratified or serial specimens, trajectory analysis and RNA kinetics to characterise tumour progression and treatment‐associated changes. Computational interpretation is followed by orthogonal and functional validation.

**TABLE 2:** Temporal strategies and limitations in spatiotemporal transcriptomics (STT).

Strategy	Temporal evidence	Supported inference	Principal limitations	Refs.
Stage‐stratified sampling	Ordered stage‐specific specimens	Population‐level stage‐associated remodelling	Inter‐patient, batch and anatomical confounding; no direct lineage or same‐cell tracking	^53^
Paired or serial clinical sampling	Same‐patient paired or serial specimens	Within‐patient remodelling during treatment or progression	Different regions and cells are sampled; sparse time points; limited biopsy feasibility	^54^
Pseudotime or spatial trajectory	Expression‐based ordering with optional spatial constraints	Putative state transitions and branching	Root and manifold assumptions; spatial adjacency is not lineage; sensitive to batch effects and spot mixing	^55^
RNA velocity or metabolic labelling	Splicing kinetics or labelled‐RNA age	Short‐term RNA dynamics and directionality	Model‐ and platform‐specific kinetic assumptions; Limited compatibility and sensitivity	^52^, ^56^
Longitudinal intravital imaging	Repeated imaging of the same field	Direct cellular behaviour over time	Limited molecular multiplexing and transcriptome coverage; depth and field‐of‐view constraints	^8^

In conventional scRNA‐seq, pseudotime orders cells along a low‐dimensional transcriptional manifold. In STT, trajectory inference must additionally account for spatial coordinates, neighbourhood structure, tissue boundaries and cross‐section deformation. Spatial adjacency may reflect paracrine exposure or shared anatomy rather than lineage, while transcriptionally similar cells can occupy disconnected regions. Results therefore depend on root‐state selection, branch structure, sampling density, spot mixing or segmentation and the method used to couple molecular and spatial distances. Pseudotime should be interpreted as a relative state ordering, not chronological time or proof that one cell population directly generated another.^55^

Samples collected at predefined or clinically documented time points allow tissue states to be compared in chronological order. However, because most spatial transcriptomic assays are destructive, serial samples generally contain different cells and often different tissue regions, rather than enabling direct longitudinal tracking of the same cells. Stage‐stratified cohorts estimate population‐level changes across disease states, whereas paired pre‐, on‐ and post‐treatment samples reduce inter‐patient confounding but usually sample different tissue regions. RNA‐velocity models add short‐term directionality from spliced and unspliced transcripts, and metabolic‐labelling approaches such as TEMPOmap measure RNA age and subcellular kinetics; both rely on model‐ and platform‐specific assumptions.^52^ Longitudinal intravital imaging remains complementary because it directly records cellular behaviour over time but profiles far fewer molecular features.^8^, ^55^

Multi‐sample integration requires two conceptually distinct operations, namely, geometric registration of sections and statistical correction of technical variation. Alignment tools such as PASTE,^57^ STAligner^58^ and SANTO^59^ seek correspondence across sections, whereas Harmony,^60^ scVI,^61^ Seurat integration,^62^ ComBat‐Seq^63^ and Crescendo^64^ address expression‐level batch effects. Aggressive correction can erase true stage‐, patient‐ or niche‐specific biology, while inadequate correction can create spurious trajectories. Performance should therefore be evaluated not only by batch mixing but also by preservation of histological domains, cell‐state diversity, spatial neighbourhoods and known stage or treatment effects. SANTO^59^ is best described as a two‐dimensional (2D) or three‐dimensional (3D) alignment and stitching method, not as a temporal‐inference algorithm.

Cell–cell communication analysis should follow, rather than substitute for, spatial and temporal modelling. CellChat^65^ and CellPhoneDB^66^ nominate ligand–receptor pairs from expression patterns; even when proximity weighting is added, predictions remain sensitive to cell segmentation or spot deconvolution, database completeness, distance thresholds, tissue boundaries and the signalling range of the ligand. RNA co‐expression does not establish protein localisation, receptor activation, directionality or causality.^67^ Contact‐dependent interactions and diffusible signals should therefore be modelled with different spatial constraints, and priority interactions should be supported by orthogonal imaging or protein measurements and, where possible, perturbation experiments.

### 3.3 Experimental designs and application boundaries

The appropriate application of STT depends on the source of temporal evidence and the strength of the claim. Stage‐stratified cross‐sectional cohorts can identify spatial programs that differ among pathological stages; paired or serial specimens can associate remodelling with treatment or progression within the same patient; and pseudotime or velocity analyses can generate hypotheses about relative state transitions. None of these designs alone demonstrates continuous cell tracking. Authors should therefore distinguish observed spatial differences from inferred trajectories and reserve causal language for experimentally validated mechanisms.^7^, ^55^

STT is particularly informative when the biological question concerns reorganisation of functional niches rather than changes in cell abundance alone. Examples include the conversion of immune‐active regions into immune‐excluded or suppressive niches, emergence of CAF‐rich invasive fronts, remodelling of perivascular and hypoxic compartments, formation of PMNs and selection of therapy‐resistant spatial states. Analyses should integrate cell composition, tissue architecture, spatially variable pathways, neighbourhood structure and temporally ordered sampling so that changes are interpreted as coordinated niche remodelling.^7^, ^68^, ^69^

Outcome‐stratified designs illustrate both the value and the limits of this framework. In high‐grade (HG) serous ovarian cancer, Ferri‐Borgogno et al. identified CAF‐rich tumour–stroma interfaces in short‐term survivors, together with candidate APOE–LRP5 communication, whereas long‐term survivors showed greater immune infiltration. This finding links a spatial niche with prognosis but does not by itself establish that APOE–LRP5 signalling caused the survival difference; the interaction remains a prioritised mechanism for validation.^70^

Archived FFPE cohorts expand access to rare tumours and longitudinal clinical material, but retrospective sampling introduces variable fixation, RNA quality, tissue area and clinical metadata. Platform compatibility with FFPE should therefore not be equated with equivalent sensitivity or whole‐transcriptome coverage across assays. Robust studies require biological replicates, harmonised sampling and processing, pathology‐guided region selection, prespecified covariates and validation in independent cohorts.^34^, ^50^

Accordingly, the application sections that follow treat STT as an inference framework rather than a real‐time monitoring technology. For each biological example, the interpretation should specify the sampled states, spatial niche, participating cell types, candidate ligand–receptor or regulatory axis, phenotype and level of validation. This structure helps separate descriptive spatial associations from supported mechanisms and clarifies which findings are ready for functional or clinical testing.

## 4 APPLICATIONS OF STT IN THE TME

Conventional high‐throughput sequencing technologies, such as bulk sequencing and single‐cell sequencing, have notable limitations in TME research, particularly regarding spatial resolution.^71^ In summary, these traditional methods cannot provide in‐depth insights into the spatial distribution, adjacency relationships and dynamic interactions of cells within the TME.

STT offers a transformative approach by integrating spatial tissue context with gene expression data. This integration enables the detailed mapping of the spatial distribution of cell types, phenotypes and functional states within tumours and their microenvironments, facilitating the reconstruction of complex cellular interaction networks.^72^ Currently, STT applications in TME research primarily focus on analysing spatial heterogeneity, uncovering temporal dynamic changes and promoting the optimisation and development of precision cancer therapies. This review will explore these aspects in depth, highlighting the significant contributions and potential of STT in advancing TME research.

### 4.1 STT in the characterisation of spatial heterogeneity in the TME

Tumour spatial heterogeneity refers to the pronounced variations in cellular composition, gene expression profiles, microenvironmental characteristics and functional states across distinct regions within tumour tissues. This heterogeneity encompasses not only the genetic and epigenetic diversity of cancer cell subpopulations but also the uneven spatial distribution and functional specialisation of non‐malignant components within the TME.^73^ Leveraging its ability to preserve spatial context, STT facilitates in‐depth analysis of this heterogeneity by characterising multicellular organisation, inferring cellular interactions, mapping spatial activity patterns of signalling pathways and identifying candidate regulatory programs associated with tumour boundaries.^74^ To illustrate the types of information obtainable from spatial profiling, Figure 3 summarises four complementary analytical dimensions in cancer tissues, namely, cell composition, cell interactions, microenvironmental organisation and microanatomy.

**FIGURE 3:**
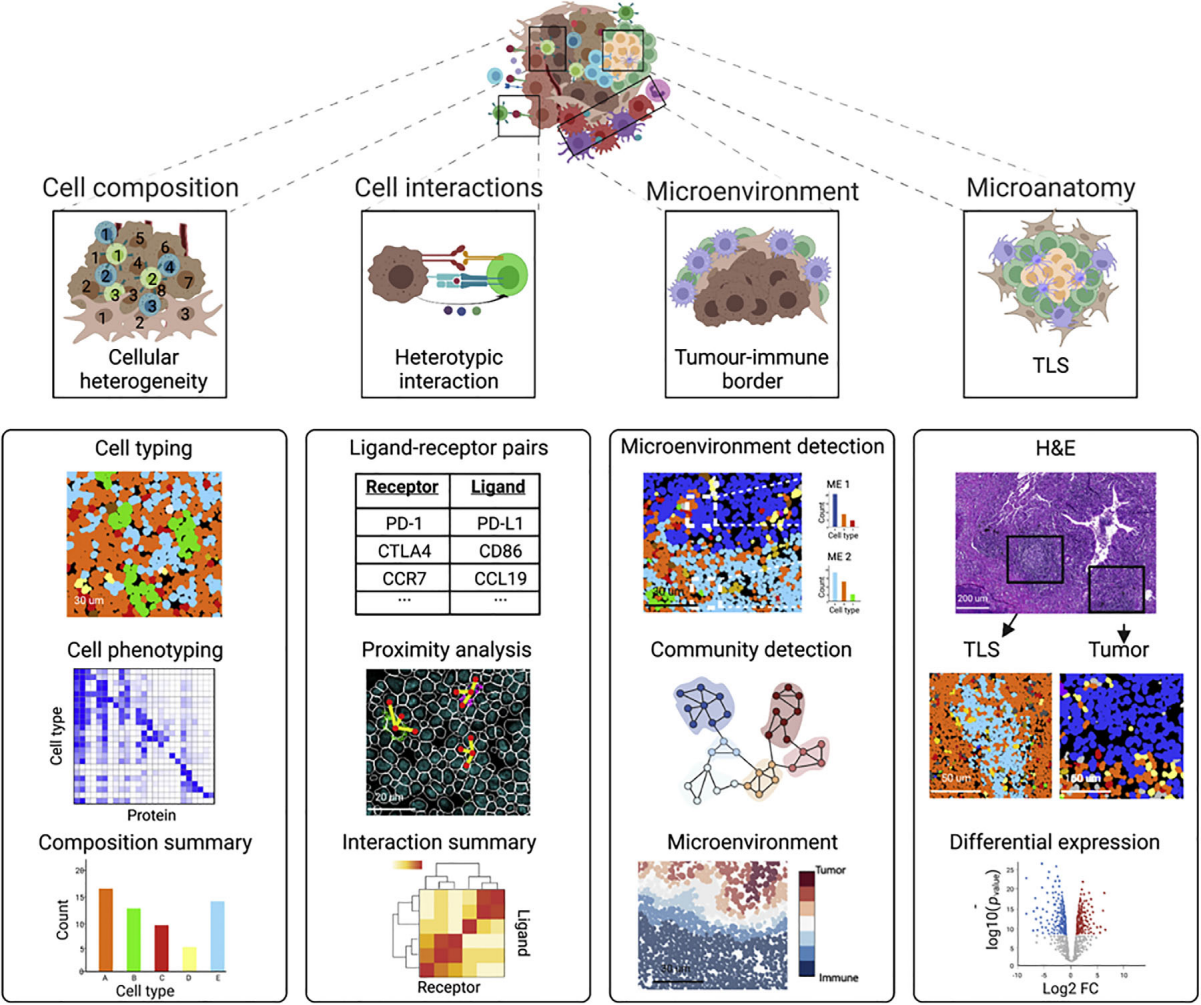
Spatial profiling in cancer research. Spatial profiling in cancer research. Spatial profiling enables the characterisation of cellular composition, intercellular interactions, microenvironmental organisation and microanatomical structures within tumour tissues. Reproduced with permission from Elsevier Publications, copyright 2023.

#### 4.1.1 Intercellular interactions

The cellular composition and functional states of the TME vary considerably depending on the tumour's organ of origin, intrinsic cancer cell characteristics, tumour stage and patient‐specific factors.^6^ By preserving spatial context, STT enables high‐resolution mapping of cellular neighbourhoods, spatial proximity and candidate intercellular communication programs during tumour initiation and progression.^69^, ^75^, ^76^ However, because most ligand–receptor analyses are inferred from transcript co‐expression and spatial adjacency, these interactions should be interpreted as spatially supported hypotheses rather than definitive evidence of functional communication.

For example, Guo et al. integrated single‐cell RNA sequencing with spatial transcriptomics to reconstruct the transition from HPV‐negative normal cervix to HPV‐infected cervix, precancerous lesions and invasive cervical cancer.^77^ Rather than simply showing a broad disruption of immune–epithelial interactions, this study suggested that epithelial–immune communication changes step by step during cervical carcinogenesis. In the normal cervix, ligand–receptor pairs such as FLG–NCAM1, C3–C3AR1, CDH1–KLRG1 and SAA1–FPR2 were associated with epithelial homeostasis, immune surveillance, tissue repair and anti‐inflammatory regulation. After persistent HPV infection, MIF–TNFRSF10D and CSF3–CSF1R/CSF3R interactions became more prominent, suggesting activation of antiviral and innate immune responses, as well as macrophage‐related immune remodelling. During progression to precancerous lesions, increased JAG1–CD46 communication indicated the emergence of immunoregulatory programs that may favour regulatory T‐cell differentiation. In invasive cervical cancer, CEACAM‐associated and WNT‐related interactions were further enriched, reflecting a shift towards tumour‐promoting and immunosuppressive epithelial–immune crosstalk. Thus, the loss of spatial homeostasis in HPV‐related cervical carcinogenesis can be understood as a gradual shift from protective epithelial and antiviral communication to immunoregulatory and malignant niche formation.

STT can also help identify ligand–receptor programs related to stromal remodelling, angiogenesis and immune suppression. In prostate cancer, Hirz et al. combined single‐cell and spatial transcriptomic analyses and showed that even low‐grade (LG) tumours can establish an immunosuppressive microenvironment.^40^ This microenvironment included suppressive myeloid populations, M2‐like macrophages, exhausted cytotoxic T cells, increased Treg activity and angiogenic stromal programs. Spatial analysis further suggested a CCL20–CCR6 communication axis between tumour‐inflammatory monocytes and Tregs, providing a possible explanation for how myeloid cells promote T‐cell suppression in the prostate TME. In addition, endothelial and perivascular cells showed tumour‐associated angiogenic transcriptional programs, indicating that stromal remodelling and vascular activation may occur early during prostate tumourigenesis. These findings show that STT is most informative when spatial localisation is linked to specific molecular axes, such as CCL20–CCR6‐mediated myeloid–Treg communication and angiogenesis‐associated stromal activation, rather than broad changes in cell distribution alone. Further validation at the protein, signalling and functional levels will help clarify the functional relevance of these inferred communication programs.

#### 4.1.2 Spatial localisation and dynamic regulation of key signalling pathways

STT can help localise signalling pathway activity to specific cell populations and spatial niches within the TME.^59^ By combining gene expression patterns with tissue architecture, this approach supports the identification of potential signal‐producing cells, responding cell populations and region‐specific regulatory programs involved in immune modulation, invasion, metastasis and therapy response. These spatially resolved signals are particularly useful for generating mechanistic hypotheses and selecting candidate targets for further validation.

In liver cancer, Wu et al. used STT to map signalling activity in the invasive tumour front, identifying a signalling cascade in which tumour‐secreted CXCL6 activates the JAK–STAT3 pathway in adjacent hepatocytes, which in turn express high levels of SAA1 and SAA2.^78^ These factors engage the TLR2/FPR1 axis to recruit and polarise macrophages towards an M2‐like phenotype, fostering an immunosuppressive niche. This region simultaneously displays enhanced EMT, lipid metabolic reprogramming, complement cascade activation and immune checkpoint expression, constituting a spatially confined signalling hub that may support local invasion.

Similarly, Bell et al. applied STT to pancreatic intraepithelial neoplasia (PanIN) and pancreatic ductal adenocarcinoma (PDAC), discovering that the progression from LG to HG PanIN is accompanied by increased expression of TFF1 and a shift from inflammatory to proliferative signalling.^79^ These changes occurred together with the spatial emergence of specific CAF subtypes and cancer stem‐like cells, highlighting their early involvement in tumour evolution. This example highlights how spatial transcriptomic analysis can connect lesion grade, epithelial state changes and stromal niche formation during early carcinogenesis.

Together, these studies show that STT is informative when pathway activity is linked to the spatial source of the signal, the responding cell type and the functional niche in which the interaction occurs. Across liver and pancreatic cancer models, spatial signalling analysis points to a common principle in which invasive or premalignant niches are shaped by coordinated communication among tumour or epithelial cells, stromal cells, parenchymal cells and immune populations. These findings provide candidate mechanisms for tumour progression and may guide spatially informed therapeutic strategies, although future functional validation will be important to test their causal roles.

#### 4.1.3 The spatial characteristics of tumour boundary maintenance

Serving as the critical interface between malignant and adjacent normal tissues, the tumour boundary is closely involved in tumour invasion, metastatic dissemination and microenvironmental remodelling.^71^ STT enables comprehensive and multi‐dimensional analyses of the cellular architecture, intercellular communication and dynamic functional transitions within these regions, while preserving spatial tissue context.^80^

In breast cancer, Janesick et al. identified a unique population of ‘border cells’ residing at the interface between tumour tissue and the myoepithelium. These cells co‐express markers from both compartments and show enrichment of genes associated with invasion and stemness, including CX3CL1, CCL28, PROM1 and KLK5, suggesting a transitional role in the progression from ductal carcinoma in situ to invasive carcinoma.^81^ In colorectal cancer (CRC), STT analyses further revealed tumour cell accumulation at the invasive front, underscoring the functional importance of spatial heterogeneity at the boundary in promoting local tumour spread.^82^

Moreover, STT allows high‐resolution mapping of subtype‐specific heterogeneity within peripheral tumour cell populations and identifies stromal–immune interaction hotspots at the tumour margin. Wang et al. utilised STT to delineate ‘endpoint‐EMT‐invasion’ gene expression programs specifically enriched in the tumour margin. These spatially confined niches, termed ‘margin invasive niches’, showed elevated expression of EMT‐related genes, revealing pronounced spatial functional heterogeneity at the tumour–immune interface.^80^ Together, these studies indicate that tumour boundaries are not passive anatomical borders, but active transition zones where epithelial plasticity, ECM remodelling and immune regulation converge. Such boundary‐focused analysis may help identify spatially restricted vulnerabilities for anti‐invasive or microenvironment‐targeted therapies.

### 4.2 Dissecting the temporal dynamic changes of the TME via STT

The TME undergoes dynamic temporal evolution throughout tumourigenesis, from precancerous lesions to primary tumour formation and eventual metastatic colonisation at distant sites. This progression is marked by continuous shifts in cellular composition, molecular signalling pathways and tissue architecture, reflecting a complex interplay across both spatial and temporal dimensions.^1^ To illustrate this spatiotemporal trajectory, Figure 4 presents a schematic overview of tumour evolution, outlining the key phases of TME remodelling during cancer initiation, progression and metastasis.

**FIGURE 4:**
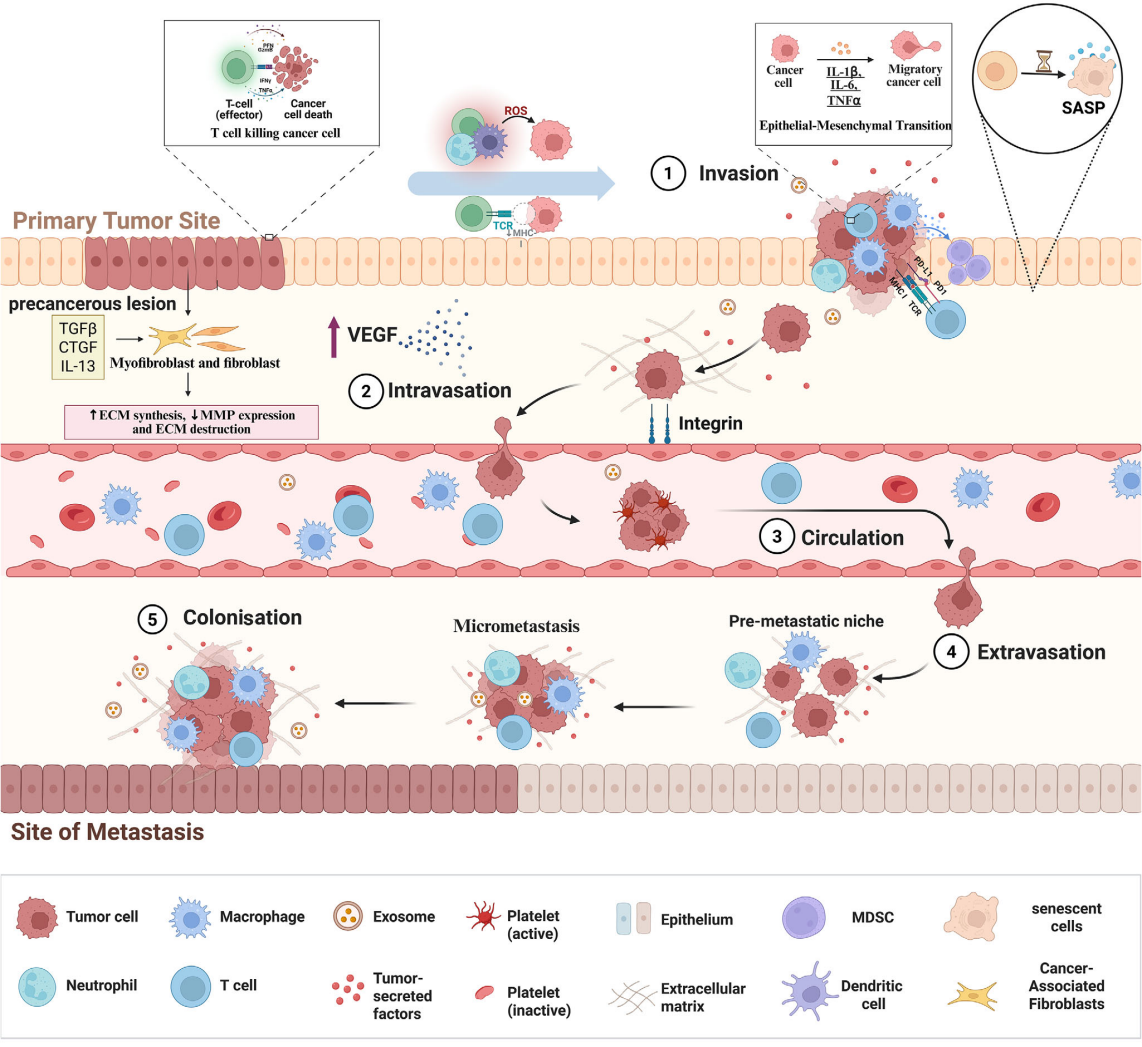
Tumour development and dynamic changes in the tumour microenvironment (TME). Tumour progression involves four critical stages: precancerous lesions, primary tumour formation, pre‐metastatic niche establishment and distant metastasis. During the precancerous stage, immune surveillance actively suppresses the expansion of abnormal cells. The primary tumour exhibits increased cellular heterogeneity, which promotes tumour growth and local invasion. The pre‐metastatic niche is remodelled through epithelial–mesenchymal transition (EMT) and the release of circulating tumour cells (CTCs), creating a favourable microenvironment for tumour cell colonisation at distant sites. Ultimately, tumour cells establish metastatic lesions in new locations, reflecting dynamic spatiotemporal evolution. Spatiotemporal transcriptomic (STT) helps characterise spatially resolved molecular programs and inferred temporal changes during tumour evolution, providing insights into tumour heterogeneity and informing precision therapeutic strategies.

#### 4.2.1 Temporal evolutionary analysis of tumour invasiveness

STT supports the integrative analysis of spatial gene expression patterns across different disease stages, providing useful clues to the cellular and molecular programs associated with tumour progression. Because most STT studies use stage‐specific samples rather than continuous tracking, they are particularly useful for comparing spatial programs across disease states and identifying regulatory events associated with cancer initiation, invasion and metastatic progression.

For instance, Sun et al. combined single‐cell RNA sequencing with spatial transcriptomics to analyse paired oral mucosal biopsies containing matched normal tissue, oral leukoplakia with moderate‐to‐severe dysplasia and early‐stage T1 oral squamous cell carcinoma (OSCC). Their analysis showed a gradual enrichment of tumour initiation‐related gene programs in epithelial cells across progressive disease states. More importantly, it indicated that OSCC initiation is accompanied by the formation of a stromal–immune niche around dysplastic epithelium. Mesen_CAFs were located close to epithelial regions and expressed ECM‐remodelling and mesenchymal‐associated genes. Spatial interaction analysis further suggested fibroblast‐associated chemokine and non‐canonical WNT communication, represented by CXCL12–CXCR4 and WNT5A–FZD6, which may support epithelial proliferation, stromal remodelling and immune‐cell recruitment. During progression from dysplasia to tumour, CAF populations expressing LOXL2, POSTN, TGFβ1 and TGFβ2 expanded, while APOE/NRG1 macrophages and TNFRSF4+ Tregs accumulated in tumour regions. Together with spatial changes in VEGFA and TGF‐β signalling, these findings indicate that OSCC initiation is shaped by coordinated epithelial–CAF–macrophage–Treg communication rather than epithelial transformation alone.^83^

Invasive growth, a hallmark of malignancy and a key step towards metastasis, remains a primary driver of cancer‐related mortality.^84^ Deciphering the spatial programs associated with invasion and treatment resistance is therefore important for understanding tumour progression and identifying potential intervention points. Kiviaho et al. used STT to characterise transcriptomic changes across prostate cancer stages, from benign tissue and localised tumours to castration‐resistant disease. They identified Club‐like epithelial cells in regions marked by low androgen signalling, inflammatory pathway activation, luminal progenitor‐like features and SASP‐related gene expression. These Club‐like regions were associated with increased PMN‐MDSC activity. Mechanistically, Club‐like cells expressed inflammatory chemokines, with CCL20–CCR6 signalling enriched at the Club–Immune interface. These findings suggest that Club‐like epithelial states may help shape a myeloid immunosuppressive niche and contribute to treatment‐resistant prostate cancer progression.^85^ This niche‐based view helps explain how spatially restricted microenvironments may support the transition from premalignant or localised lesions to invasive or treatment‐resistant disease.

#### 4.2.2 Dynamic remodelling of the immune microenvironment: Fostering immunosuppression and immune evasion

STT enables high‐resolution mapping of gene expression within specific cellular subsets or localised TME regions, thereby helping to characterise immune remodelling across disease stages and treatment conditions.

For example, Wu et al. analysed paired pre‐ and post‐neoadjuvant chemotherapy (NAC) liver metastasis samples from CRC patients. They identified a metabolically hyperactive MRC1^+^CCL18^+^ M2‐like macrophage subset with immunosuppressive properties enriched in pre‐treatment lesions. In NAC responders, this subset markedly declined, accompanied by expansion of GZMK^+^XCL1^+^ cytotoxic CD8^+^ T cells, indicating immune reactivation. In contrast, non‐responders showed persistent or exacerbated immunosuppression.^86^ This study illustrates how STT deciphers spatial immune remodelling under therapeutic pressure and highlights metabolic reprogramming as a promising intervention target.

Furthermore, STT contributes to understanding the PMN, a transient, tumour‐primed microenvironment in distant, metastasis‐free organs mediated by tumour‐derived secretions and extracellular vesicles. Preventing PMN formation may reduce metastasis risk and improve immunotherapy efficacy.^87^ Liu et al. identified a subset of epithelial cells with elevated nucleotide metabolism (NUhighepi), spatially co‐localised with fibroblasts and endothelial cells in signalling‐active regions. These cells exhibited enhanced stemness, migratory potential, immune evasion and activated key pathways such as COL1A1–ITGB1 and MIF–TNFRSF14, contributing to matrix remodelling and inflammatory regulation.^31^ Their findings suggest that NUhighepi cells may participate in establishing a metastasis‐permissive niche.

Collectively, these studies underscore STT's critical role in revealing dynamic spatial immune landscapes and early metastatic cues, offering novel insights for precision therapeutic strategies and prognostic assessment.

#### 4.2.3 Temporal evolution of metabolic adaptation in the TME

Cancer cells adapt to nutrient‐deprived conditions by reprogramming the metabolism of surrounding non‐malignant cells. This adaptation continuously reshapes the metabolic landscape of the TME, characterised by hallmark alterations such as hypoxia, acidity and lactate accumulation.^88^ These changes collectively promote the polarisation of immunosuppressive cells and the release of immunoregulatory molecules, further reinforcing a tumour‐promoting microenvironment.^89^

Liu et al. used STT together with single‐cell analysis to investigate nucleotide metabolism in CRC.^31^ They identified a tumour epithelial subset with elevated nucleotide metabolism, termed NUhighepi, which showed enhanced stemness, migratory potential, immune‐evasion features and stronger interactions with fibroblasts and endothelial cells. Spatial trajectory analysis further suggested that regions with high nucleotide metabolism were associated with stronger metastatic potential during intratumoural progression. Candidate communication axes such as COL1A1/COL1A2–ITGB1 linked NUhighepi cells with matrix remodelling and stromal interaction, while NME1 was highlighted as a potential metastasis‐suppressive factor. These findings suggest that STT can connect metabolic heterogeneity with spatial niche remodelling and inferred progression trajectories.

STT can also help characterise lactate‐associated metabolic remodelling in the TME. For example, in OSCC, Liu et al. applied STT to elucidate how tumour‐derived lactate shapes TME remodelling via a HIF1A‐centred metabolic–signalling–immune‐regulatory axis.^90^ This cascade links lactate accumulation with downstream immune‐regulatory programs, suggesting that metabolic reprogramming and immune suppression may be spatially coordinated. These findings provide a rationale for combination strategies that target both metabolic and immune pathways.

#### 4.2.4 Temporal analysis of tumour drug resistance mechanisms

Drug resistance remains one of the greatest challenges to achieving a cure for advanced cancers.^91^ STT can map resistance‐associated transcriptional programs within tumour tissues and their surrounding microenvironment, helping to identify spatial relationships among resistant tumour cells, immune cells and stromal cells.^92^ When applied to paired pre‐ and post‐treatment samples or longitudinal tumour models, STT provides spatially resolved clues to how resistant niches emerge, expand and interact with the TME during therapeutic pressure.

Kats et al. employed STT to demonstrate that drug resistance mechanisms in chromothripsis‐type SHH medulloblastoma exhibit a multiclonal origin and spatial heterogeneity. Tracking treatment progression in patient‐derived xenograft models, they found that recurrent tumours are driven by multiple genetic clones coexisting spatially, forming an ecological system. These clones establish intercellular networks through tumour microtubes and collaboratively transmit calcium signals to enhance treatment resistance. Activation of pathways such as Midkine in specific clones further exacerbates drug resistance.^93^ This work illustrates STT's capability to analyse the formation of drug resistance, particularly by revealing the spatial localisation, transcriptional states and dynamic interactions of drug‐resistant cell populations within the microenvironment.

Therapeutic resistance is closely associated with EMT and CTCs.^94^ EMT, a key driver of tumour metastasis, involves hybrid cells exhibiting both epithelial and mesenchymal characteristics, possessing high plasticity and undergoing critical state transitions during intravasation, circulation and distant colonisation.^33^ A deeper understanding of the molecular mechanisms of EMT and its interplay with CTCs in mediating drug resistance is of great significance.^94^ Wang et al. mapped the evolutionary trajectory of tumour cells from invasive fronts to stable states by identifying progressive gene expression changes throughout EMT from early to late cancer stages.^80^ By linking EMT‐associated plasticity with spatial tumour architecture, STT may help identify vulnerable niches for strategies aimed at limiting invasion, metastasis and resistance.

#### 4.2.5 Remodelling and reconstruction of the TME during the aging process

Systemic aging is a major risk factor for cancer, primarily due to the cumulative effects of diverse molecular and cellular processes over time. The impact of aging on tumour progression is stage‐dependent. During early tumour development, aging can suppress tumour growth by inducing cell cycle arrest and enhancing immune surveillance, whereas at later stages, it may promote tumour progression by triggering chronic inflammation and immunosuppression.^95^ Specifically, aging influences the TME mainly through alterations in the distribution and function of immune and stromal cells. Various immune cell types contribute to immunosuppression and tumour progression within the TME, accompanied by spatiotemporally specific changes in gene expression.^96^

STT enables the elucidation of temporal correlations and interactions that govern molecular dynamics within the complex aging network. Moreover, STT can reconstruct trajectories of disease‐associated state changes; comparative analyses of spatiotemporal multi‐omics data from healthy and diseased tissues clarify aging‐related changes in the TME and provide precise insights into underlying mechanisms. The senescence‐associated secretory phenotype (SASP) is a key mechanism by which senescent cells influence the microenvironment via paracrine and endocrine signalling.^97^ SASP components include pro‐inflammatory factors, proteases and growth factors, exhibiting dual biological effects with spatiotemporal specificity. Prince et al. utilised STT to characterise the spatial and temporal features of SASP in adamantinomatous craniopharyngioma. They found that SASP‐positive cells extend beyond traditional epithelial clusters and are also widely distributed within palisading epithelium and glial regions, thereby establishing a localised senescent microenvironment. Through pseudotime modelling, the study further demonstrated dynamic activation of the PI3K/Akt/mTOR, MYC and Hedgehog pathways during senescence, alongside progressive attenuation of p53‐regulated signalling. These findings underscore STT's unique capacity to resolve senescence‐associated cellular heterogeneity and spatiotemporal dynamics, offering critical insights for therapeutic strategies targeting the senescent microenvironment.^98^

Compared with bulk transcriptomics and dissociated single‐cell approaches, STT not only preserves spatial context but also links age‐ or stage‐associated transcriptional changes to specific tissue neighbourhoods. Although temporal information is usually inferred from age‐stratified samples or pseudotime analysis rather than real‐time tracking, this approach helps clarify how aging reshapes immune–epithelial–stromal interactions, inflammatory states and gene‐expression programs within tissue microenvironments. Angarola et al. applied single‐cell multi‐omics and STT to mammary tissues from young and aged C57BL/6J mice. They found age‐related changes in epithelial, stromal and immune cell states, with spatial transcriptomics showing increased co‐occurrence of aged immune‐cell markers near epithelial regions. Candidate ligand–receptor analyses further suggested age‐associated integrin‐mediated interactions and Jag1–Notch signalling among immune, epithelial and stromal populations. These findings provide spatially resolved clues linking aging‐associated tissue remodelling with inflammatory and potentially tumour‐prone microenvironments, offering potential entry points for interventions targeting aging‐related cancer susceptibility.

### 4.3 STT drives precision oncology

STT supports precision oncology by linking spatially organised cellular states and molecular programs to disease progression, therapeutic response and potential therapeutic vulnerabilities. Across cancer types, STT studies have identified candidate targets involving tumour cells, immune populations, stromal components and intercellular signalling pathways. Representative applications and the corresponding potential therapeutic targets are summarised in Table 3.

**TABLE 3:** Applications of spatiotemporal transcriptomics (STT) in various cancer types and summary of potential therapeutic targets.

Tumour types	Platform	Target	Significance	Refs.
PCa	Slide‐seqV2	TIMo; M2‐MΦ; CCL20‐CCR6; VEGFA	Deciphering the immunosuppressive microenvironment; Revealing the association between cellular interactions and angiogenesis	^40^
PDAC	Stereo‐seq	EDB‐FN1; IL‐2R	Deciphering the microenvironment; Validate immune activation	^99^
CRC	Visium HD and Xenium	SELENOP; SPP1; CXCL9/10/11	Deciphering spatial interactions of immune cells in the microenvironment	^100^
CSCC	Stereo‐seq	myCAFs; LGALS9; IDO1	Deciphering the microenvironment; myCAFs association with tumour progression	^101^
HCC	Stereo‐seq	SAA1/2; CXCL6; JAK‐STAT3; FPR1; TLR2	Deciphering the 500 µm invasive zone at the tumour margin; Revealing mechanisms of immune suppression and metabolic reprogramming	^78^
NPC	Stereo‐seq and Visium	CXCL13; VCAM1	Deciphering TLS; Promoting B cell activation and immunotherapy	^102^
ccRCC	Visium and GeoMx and CosMx	IL‐1β; LAG3; HAVCR2	Deciphering spatial heterogeneity of the microenvironment; Revealing interactions among EMT, metabolism and immunity	^103^
FL	Visium and GeoMx	CXCL13; DC‐SIGN;	Microenvironment analysis, Reveal cell–cell interactions and matrix remodelling	^104^
LUAD	Visium	IDO1; SPP1; CXCL14; CAFs	Deciphering TME interactions and progression mechanisms	^53^
Medulloblastoma	Stereo‐seq	SOX4; PTPRZ1	Deciphering spatial heterogeneity and evolutionary trajectories	^105^
BC	Slide‐seq and MERFISH	CD163; SOX4; MMP11; VIM	Deciphering microenvironment interactions; Identifying the association between EMT and immunity	^106^
NSCLC	Visium and GeoMx and MERFISH	CXCL10; CCL19; TCF7; PD‐1	Deciphering the immune microenvironment; Identifying stem cell–immune hubs	^107^

#### 4.3.1 Mining cellular and molecular targets based on spatiotemporal dimensions

Compared with bulk transcriptomics or dissociated single‐cell approaches, STT preserves spatial tissue context and links molecular programs to specific cellular neighbourhoods. Although intravital imaging is better suited for directly tracking cellular behaviours such as migration, invasion and treatment response over time, STT adds transcriptome‐wide spatial information that helps connect these dynamic behaviours with candidate molecular programs. This complementary advantage makes STT particularly useful for prioritising context‐specific therapeutic targets within the TME.

Unlike conventional differential‐expression analyses, STT can prioritise candidate targets according to not only expression magnitude but also spatial localisation, cell‐type specificity, association with defined tissue niches and changes across disease stages or treatment conditions. A molecule becomes particularly informative when it is enriched within a biologically relevant compartment, such as an invasive front, immune‐excluded region, perivascular niche or therapy‐resistant cluster and is linked to a coherent cellular or signalling program.^108^ Additional support may come from consistent associations with progression or treatment response, participation in plausible ligand–receptor or signalling networks and reproducibility across patients or independent datasets.

These spatial and contextual features can therefore help distinguish potentially informative candidates from broadly expressed differentially expressed genes and guide subsequent mechanistic investigation. Such candidates should initially be regarded as spatially supported hypotheses, with their biological and therapeutic relevance established through orthogonal and functional validation.^109^ Figure 5 summarises the regulatory influences and biological functions of the TME, highlighting how cellular, spatial and temporal context can inform candidate target identification and therapeutic interpretation.

**FIGURE 5:**
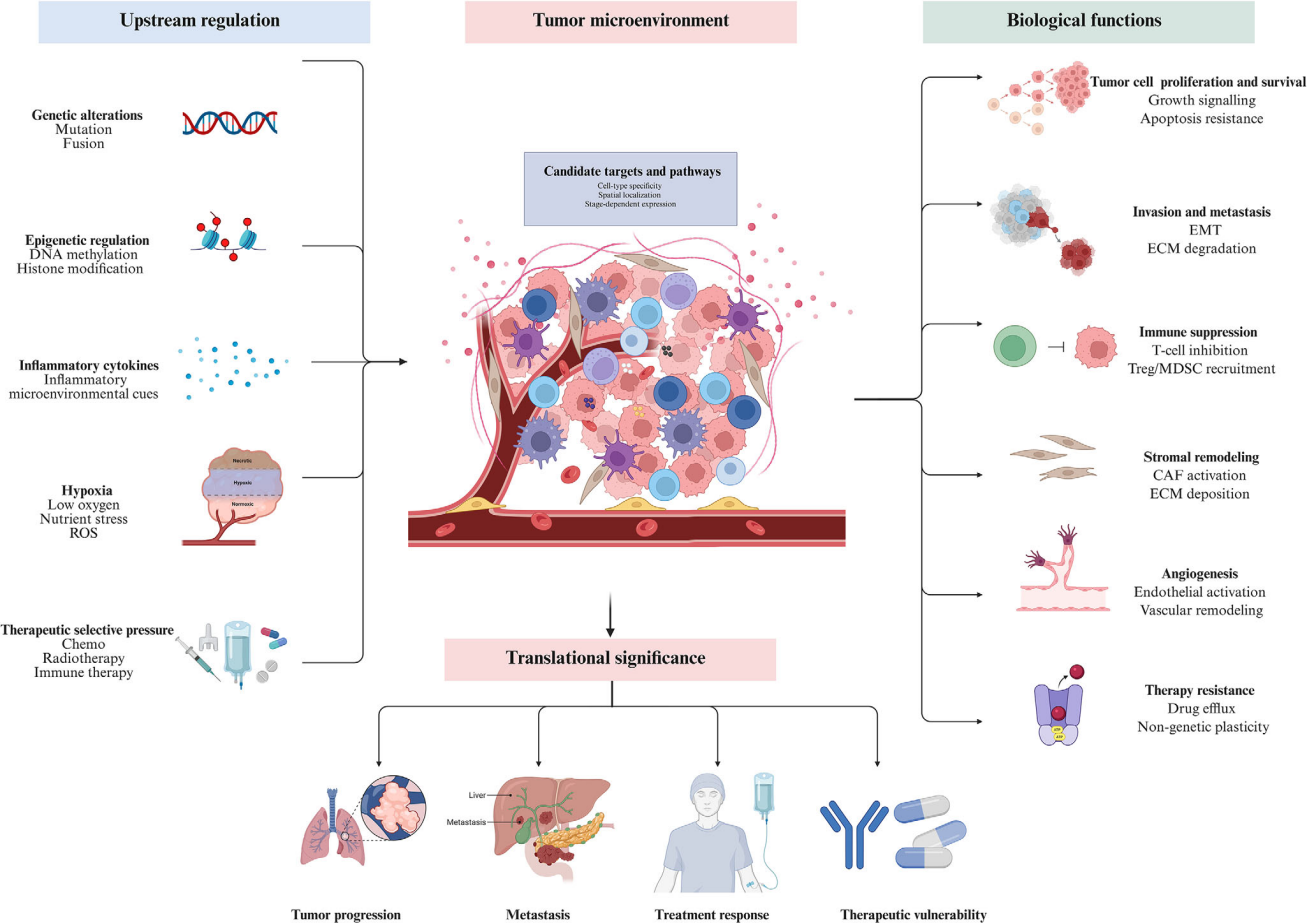
Regulation and functional significance of the tumour microenvironment (TME). Genetic and epigenetic alterations, inflammatory signals, hypoxia and therapeutic selective pressure shape molecular programs within the TME. These programs influence tumour growth, invasion, immune suppression, stromal remodelling, angiogenesis and therapy resistance. Their cellular, spatial and temporal features inform candidate target identification and the interpretation of tumour progression and treatment response.

STT enables detailed characterisation of the functional roles and intercellular interactions of distinct cellular subpopulations during tumour progression, supporting the reconstruction of gene regulatory networks and signalling pathways within the TME. This, in turn, facilitates the identification of precise, context‐specific therapeutic targets for personalised intervention.^110^ Agostini et al. applied STT to investigate HG) and LG intraductal papillary mucinous neoplasms (IPMNs), uncovering mucin‐specific O‐glycosylation as a top differentially regulated gene program both between LG and HG IPMNs and during the transition to invasive carcinoma.^111^ They further identified GCNT3 as a key marker of malignant progression, highlighting its therapeutic potential in pancreatic cancer. Similarly, Sans et al. identified NKX6‐2 as a critical transcriptional regulator of gastric cell fate in LG IPMNs, suggesting its utility as a biomarker for low‐risk lesions.^112^ Together, these studies suggest that STT can help define spatially resolved molecular programs and candidate regulators associated with tumour evolution across disease stages.

Beyond target discovery, STT has revealed the spatially heterogeneous distribution of tumour clones across various solid tumours, where functionally distinct subclonal populations form specialised microniches through direct cell–cell interactions and paracrine signalling. For instance, in medulloblastoma, a multiclonal ecosystem was shown to collectively resist therapy and promote recurrence via a tumour microtube network.^93^ These observations suggest that disrupting clonal communication networks may offer a novel strategy to overcome treatment resistance.

Furthermore, STT can help identify immune‐exclusion barriers and spatially restricted immunosuppressive niches within the TME, supporting the development of combination therapies that target multiple cellular and molecular states. Collectively, these insights highlight the translational value of STT in precision oncology, particularly for prioritising spatially informed, multi‐target therapeutic strategies.

#### 4.3.2 Spatial context and temporal dynamics of representative candidate targets and pathways

The biological relevance of candidate targets and pathways can change across disease progression and treatment.^93^ Molecular programs associated with early adaptation may differ from those that characterise residual disease or acquired resistance, while their functional relevance can also depend on the spatial cellular context in which they operate.^113^ Integrating treatment‐stage sampling with spatial information can therefore help identify when and where candidate molecular programs become relevant.

In BRAF‐mutant melanoma, Rubinstein et al. applied spatial transcriptomics across multiple treatment time points in patient‐derived xenograft models to profile tumour evolution from pre‐treatment growth through drug‐tolerant persistence and resistant regrowth during combined BRAF and MEK inhibition. Persister populations showed reduced proliferation, increased oxidative phosphorylation and increased invasive capacity, together with spatial differences between central and peripheral tumour regions. MAPK feedback regulators also displayed distinct lineage‐ and stage‐dependent patterns. DUSP4 was suppressed in resistant but not sensitive lineages in one model, whereas several DUSP family members decreased during early treatment and were re‐expressed in late‐arising proliferative lineages in another.^113^ Multi‐time‐point spatial profiling, together with phylogenetic tracing, therefore highlighted MAPK feedback regulation as a candidate process whose relevance changes during adaptation and resistance. This example illustrates how temporal sampling and spatial context can refine target interpretation beyond a single treatment endpoint.

#### 4.3.3 Genetic and epigenetic regulation and functional validation of spatially characterised candidate targets

Spatially characterised candidate targets require complementary functional and regulatory analyses to determine whether they actively contribute to the associated phenotype. Genetic perturbation can test functional involvement, whereas epigenomic analyses can reveal regulatory mechanisms that establish or maintain treatment‐associated cellular states.^114^

Zhang et al. provided an example of genetic validation in PDAC. CD276/B7‐H3 was prioritised because its expression increased after chemotherapy and localised to a chemoresistant niche containing SNCG^+^ basal‐like tumour cells, SPP1^+^ macrophages and exhausted T cells. CRISPR–Cas9 knockout enhanced CD8^+^ T‐cell‐mediated tumour killing, while B7‐H3 blockade in KPC tumours shifted malignant‐cell programs away from the basal‐like state and reduced immunosuppressive and angiogenic programs.^115^ These findings support a functional role for B7‐H3 in both tumour‐cell plasticity and immune dysfunction, providing a rationale for therapeutic strategies that combine B7‐H3 targeting with chemotherapy.

A complementary example of epigenetic regulation was reported by Veo et al. in therapy‐resistant Group 3 medulloblastoma. Integrated single‐nucleus RNA and ATAC profiling, together with spatial validation, identified expansion of a progenitor‐like resistant state associated with chromatin and metabolic reprogramming. Radiation‐resistant cells showed altered H3K4me3, H3K27ac and H3K27me3 landscapes, with wild‐type IDH1 implicated as a regulator of this state. IDH1 suppression partially reversed resistance‐associated chromatin changes, while pharmacological inhibition increased radiosensitivity and shRNA‐mediated depletion improved outcome in orthotopic models.^114^ These results connect a spatially characterised resistant state with an experimentally supported metabolism–epigenetic regulatory mechanism.

Together, these examples illustrate complementary approaches to target validation. CRISPR‐mediated perturbation can establish functional involvement of a candidate molecule, whereas epigenomic profiling combined with targeted perturbation can reveal mechanisms that regulate treatment‐associated cell states.

#### 4.3.4 Immunotherapy optimisation: Targeting the spatiotemporal dynamics of the immune microenvironment

Compared to traditional cancer treatments, immunotherapy offers new therapeutic opportunities and has emerged as a promising strategy in contemporary oncology research.^116^ STT enables comprehensive comparisons of the TME before and after treatment, providing a robust foundation for guiding personalised cancer therapy. Tang et al. employed STT to precisely map the spatial distribution and developmental states of TLSs within the TME, revealing that tumour cells induce TLS immaturity and suppress local immunity through enhanced tryptophan metabolism. Inhibition of this pathway was shown to promote TLS maturation, enhance immune responses and synergise with anti‐PD‐1 therapy. Based on these findings, the authors proposed a novel immunotherapeutic strategy combining anti‐PD‐1 treatment with tryptophan metabolism inhibitors and suggested that TLS maturation status could serve as a predictive biomarker for immunotherapy responsiveness and patient stratification.^109^

STT also contributes to guiding precision immunotherapy by identifying spatially restricted immune landscapes and identifying candidate mechanisms of therapeutic resistance while preserving spatial context.^117^ Ji et al. applied STT to investigate cutaneous squamous cell carcinoma and identified tumour‐specific keratinocytes (TSKs) predominantly localised at the tumour margins. These cells formed a fibrovascular niche co‐localised with Tregs, CD8^+^ T cells and DCs expressing PD‐L1/PD‐L2, collectively establishing an immunosuppressive microenvironment. Further analyses demonstrated that elevated expression of TSK marker genes such as ITGB1 and PLAU correlates with immunotherapy resistance and poor clinical prognosis.^108^ Through an integrated research framework of ‘spatial localisation–mechanistic analysis–targeted intervention’, STT, combined with complementary spatiotemporal approaches, offers novel insights into overcoming immunotherapy resistance and elucidating the dynamic evolution of the TME.

#### 4.3.5 Spatiotemporal analysis applications in personalised treatment strategies

Integrating STT with single‐cell sequencing and other complementary modalities may improve patient stratification and support personalised cancer diagnosis and treatment. By comparing normal tissues, precancerous lesions, early‐stage tumours and advanced malignancies, STT can reconstruct disease‐associated spatial changes and identify candidate biomarkers for early detection and intervention.^1^ STT also enables comparisons of spatial and functional heterogeneity between primary tumours and metastatic sites, thereby revealing spatial ecotypes with potential therapeutic relevance.

For instance, Khaliq et al. employed STT to identify that primary tumours enriched in myofibroblasts, immunosuppressive macrophages and Tregs, notably within spatial ecotypes such as CC1 and CC5, exhibited pronounced fibrosis and immunosuppression. These characteristics suggest resistance to conventional immunotherapy, indicating a potential benefit from combined anti‐fibrotic or CAF‐targeted therapies. In contrast, the CC3 ecotype, predominantly found in liver metastases, was characterised by high tumour cell proliferation and enhanced glycolytic activity, suggesting responsiveness to glycolysis inhibitors or other metabolic intervention strategies. Additionally, the immune‐enriched CC7 ecotype, located at the invasive tumour margins, consisted of CD8^+^ T cells, M1‐like macrophages and Tregs, forming an ‘immune tug‐of‐war’ state that may represent a region responsive to immune checkpoint inhibitors.^118^ These findings underscore STT's dual utility in both patient stratification and the selection of microenvironment‐specific therapeutic strategies, ultimately advancing the realisation of personalised precision oncology.

Despite these promising findings, the evidence supporting many STT‐derived therapeutic targets remains preliminary. Only a minority of the target‐oriented studies cited in this review incorporated direct functional validation; most targets were instead nominated primarily through computational or spatial association analyses. Consequently, many STT‐derived targets remain unproven therapeutic hypotheses that require rigorous experimental validation.

## 5 NEW DIRECTIONS IN STT RESEARCH

Building on the applications and methodological limitations discussed above, the next phase of STT research will require coordinated advances in measurement technologies, computational analysis and clinical validation. Three directions are particularly important extending spatial profiling from individual 2D sections to 3D and multimodal representations, developing standardised and reproducible analytical frameworks and translating spatiotemporal features into clinically validated biomarkers and therapeutic strategies. These emerging directions are discussed below.

### 5.1 Innovative advances in technology and data analysis methods

Structures that appear confined or localised in conventional 2D imaging often reveal complex, hierarchical and interconnected architectures when visualised in three dimensions. This multilayered spatial organisation not only provides the physical substrate for interactions between tumour and immune cells but also constitutes a crucial framework underpinning immune evasion, cellular migration and the development of intratumoural heterogeneity.^119^ Recent advances in ST have facilitated the extension of 2D spatial gene expression data into 3D reconstructions, enabling a more comprehensive and volumetric analysis of tissue architecture and microenvironmental dynamics. By integrating ST with tissue‐clearing, serial‐sectioning and image alignment techniques, researchers have achieved single‐cell‐resolution molecular profiling across entire organs and even whole embryos, capturing both spatial and temporal dimensions. For instance, applications of 3D‐ST in mouse embryogenesis have elucidated developmental trajectories and unveiled the spatial regulatory networks driving organogenesis.^120^, ^121^ These pioneering studies highlight the potential of 3D‐ST to unravel fundamental biological processes.

Although many applications of 3D spatial transcriptomics remain focused on developmental biology, recent experimental and computational methods have expanded its feasibility in complex tissues. Open‐ST^72^ provides a scalable approach for high‐resolution spatial transcriptomic profiling and 3D tissue reconstruction, whereas SANTO^59^ supports the alignment and stitching of spatial omics sections. In cancer research, these approaches may improve the reconstruction of tumour–stroma interfaces, immune barriers, vascular structures, invasive fronts and metastatic routes. Further technical optimisation should focus on tissue preservation, serial‐section alignment, correction of tissue deformation, cell segmentation, molecular sensitivity, throughput and reproducibility across samples.

Integration with complementary technologies represents another important direction. Despite the spatial advantages of current spatial transcriptomic platforms, trade‐offs remain among detection sensitivity, gene coverage, spatial resolution and tissue area.^122^ Integration with scRNA‐seq can improve cell‐type annotation and facilitate the spatial localisation of rare or transitional cell states within the TME.^123^, ^124^ Complementary approaches discussed in the Technical Principles section, including intravital imaging and spatial protein analysis, can further provide dynamic or protein‐level evidence for validating cellular trajectories and interactions inferred from STT data.

More broadly, spatial transcriptomics alone cannot capture all molecular features underlying tumour evolution. Integration with genomics, epigenomics, proteomics, metabolomics and immunomics may provide a more comprehensive view of cellular identity, regulatory state and microenvironmental interactions. For instance, Sun et al. conducted an integrative multi‐omics study of patients with metastatic hepatocellular carcinoma (HCC), incorporating genomic, transcriptomic and immunomic datasets to delineate the evolutionary trajectories of metastasis. Their findings revealed that hypoxia‐enriched primary tumours exhibit early multiclonal dissemination, with pronounced genomic divergence between primary and metastatic lesions, indicating early metastatic seeding. Somatic copy number alterations emerged as key drivers of metastatic expansion. At the immune level, high neoantigen heterogeneity and compromised antigen presentation in metastatic lesions were associated with diminished T‐cell responsiveness, emphasising the role of immune escape in metastatic evolution. Moreover, specific subclones lacking Wnt pathway mutations exhibited enhanced metastatic potential, residing within a microenvironment enriched for activated fibroblasts and immunosuppressive B cells. These B cells facilitated terminal CD8^+^ T‐cell exhaustion via the HLA‐E–CD94‐NKG2A axis.^125^ Collectively, this study provides a multi‐dimensional framework for understanding metastatic HCC and establishes a foundation for subsequent validation of key cellular subsets and microenvironmental interactions through spatial transcriptomic techniques.

### 5.2 Algorithm improvement

Future algorithm development should be accompanied by standardised, reproducible analytical workflows for STT. These workflows should provide clear recommendations for data quality control, spatial‐domain identification, cell‐type annotation, trajectory inference, cell–cell communication analysis, multi‐sample integration and result validation. Minimum reporting requirements should also be defined for sample metadata, pre‐processing parameters, spatial‐neighbourhood construction, batch‐correction strategies and statistical thresholds, thereby improving transparency and comparability across studies.

For trajectory analysis, future frameworks should integrate transcriptional states, spatial organisation and sampling stages to reconstruct cellular and tissue‐level changes during tumour progression and treatment. Because inferred trajectories can be influenced by spatial constraints, starting‐state selection and uneven sampling, recommended analytical practice should include sensitivity analyses using alternative parameters or methods and comparison with pathological stages, anatomical structures or treatment time points.^32^ Such procedures would help distinguish robust biological patterns from results that depend strongly on a particular analytical setting.

Cell–cell communication analysis should likewise evolve from the generation of ligand–receptor lists towards the identification of spatially organised signalling programs. Future methods should incorporate cellular neighbourhoods, tissue compartments, histological boundaries and the spatial range of different signalling modes. Standardised workflows should report how neighbourhoods and distance thresholds are defined and should prioritise interactions that are consistently detected across analytical approaches or supported by imaging, protein‐level measurements, or functional experiments.

Multi‐sample integration and batch correction will be particularly important for studies involving different patients, tissues, disease stages, treatment time points and experimental platforms. However, many existing batch‐correction algorithms were originally developed for scRNA‐seq and remain insufficiently adapted to heterogeneous STT datasets spanning multiple tissues and extended disease or treatment timelines, because they do not explicitly model spatial architecture, temporal ordering and tissue‐specific biological variation. Future analytical strategies should use detailed experimental and clinical metadata to model major sources of variation while preserving biologically meaningful spatial differences. Benchmark datasets with known tissue structures and controlled technical variation would facilitate objective comparison of integration methods. Evaluation should consider not only the removal of batch effects but also the preservation of spatial domains, cell‐state diversity and stage‐ or treatment‐associated changes.

For 3D reconstruction and multi‐omics integration, future algorithms should provide standardised procedures for section registration, alignment‐quality assessment, resolution matching and the integration of partially overlapping molecular features. Together with accessible software, shared benchmark datasets and well‐documented analysis pipelines, these developments would make STT analyses more robust and facilitate their application by both experienced researchers and investigators entering the field.

### 5.3 Clinical application

Future clinical studies should move beyond descriptive spatial atlases and use STT to address defined questions concerning treatment response, resistance and biomarker development. In cancer immunotherapy, prospective analysis of pre‐treatment, on‐treatment and progression samples could characterise how tumour–immune interfaces, immune‐excluded regions, TLSs, antigen‐presentation programs and exhausted T‐cell states change during therapy. Comparisons between responders and non‐responders may support the development of spatial signatures associated with treatment sensitivity, primary resistance or acquired resistance and help identify microenvironmental features that warrant further functional investigation.

A similar strategy could be applied to metabolism‐targeted therapies by examining how metabolism‐related transcriptional states and their spatial relationships with tumour, stromal and immune cells change following treatment. Integration with spatial metabolomics, imaging or functional measurements could further connect these transcriptional patterns with regional metabolic changes and clarify their association with therapeutic response or adaptation.

Translation into clinical practice will require prospective, adequately powered cohorts; standardised sampling time points; multi‐region tissue collection; detailed pathological annotation; and integration with treatment response and survival data. Spatial biomarkers should be validated in independent and multicentre cohorts, assessed for reproducibility across platforms and tissue‐processing protocols, and evaluated against established histopathological, molecular and clinical indicators. In parallel, simplified and cost‐effective assays will be needed to transfer clinically informative spatial features from discovery‐scale platforms into routine pathological assessment.

## 6 CRITICAL VIEW

Although spatial transcriptomics technologies have substantially expanded the ability to characterise tumour ecosystems, several conceptual and technical limitations continue to restrict their broader biological and clinical application. Most current studies remain largely exploratory, because temporal information is often inferred indirectly from cross‐sectional spatial datasets rather than captured through true longitudinal sampling. As a result, the dynamic processes underlying tumour initiation, progression, metastasis and therapeutic response remain incompletely resolved. Technical factors, including sample preparation, throughput, spatial resolution, segmentation accuracy, batch effects and computational inference, further limit reproducibility and cross‐study comparability.

Despite these limitations, STT provides a unique and non‐redundant capability by combining broad molecular profiling with preserved tissue architecture, thereby assigning molecular programs to specific cell populations, cellular neighbourhoods, histological boundaries and functional niches. Longitudinal intravital imaging enables direct same‐cell tracking and temporal continuity but is generally constrained by molecular multiplexing, imaging depth and field of view. In contrast, STT usually cannot continuously track the same cells because tissue profiling is destructive, but it provides substantially broader spatial molecular information across multiple tissue compartments. The two approaches should therefore be integrated, with intravital imaging capturing cellular behaviour over time and STT identifying the molecular programs and spatial niches associated with those behaviours.

A major analytical challenge concerns the interpretation of cell–cell communication analyses. Widely used ligand–receptor inference frameworks, such as CellChat and CellPhoneDB, were originally developed for single‐cell RNA sequencing data and do not fully account for spatial proximity, tissue architecture, neighbourhood context or the physical constraints of cell interaction. Therefore, inferred ligand–receptor pairs should be interpreted as hypothesis‐generating signals rather than definitive evidence of functional communication within the TME.

In this context, this review aims to complement existing spatial transcriptomics literature by highlighting the temporal aspects of TME remodelling. We discuss how stage‐specific samples, treatment‐related comparisons, trajectory inference, spatial neighbourhood analysis and computational reconstruction strategies may help infer potential spatiotemporal changes in tumour tissues. Particular attention is given to tumour initiation, progression, metastasis and therapy response, together with the methodological limitations that should be considered when interpreting these analyses.

Taken together, integrating STT technologies with systems‐level analytical frameworks may provide new opportunities to investigate tumour evolution, microenvironmental remodelling and therapy‐associated adaptation in a spatially resolved manner. These advances may support more mechanistic interpretations of tumour progression and treatment response, while also highlighting the need for further methodological refinement and experimental validation.

## AUTHOR CONTRIBUTIONS

**Ying Kang** and **Qinjing Wang** designed the content and wrote the first draft; **Siqi Xu**, Ying Kang and **Jiming Guo** produced the figures; **Yue Ming**, **Min Wang** and **Yisheng Huang** produced the table; Ying Kang, **Weixing Chen**, Jiming Guo and **Zhijie Huang** revised the manuscript. **Bo Jia** was responsible for supervision of the entire project as the corresponding author.

## CONFLICT OF INTEREST STATEMENT

The authors declare no conflicts of interest. Graphical abstract, Figure 1, Figure 2, Figure 4 and Figure 5 were created with BioRender.com.

## ETHICS STATEMENT

Authors declare human ethics approval was not needed for this study.

## CONSENT TO PARTICIPATE

Not applicable.

## CONSENT FOR PUBLICATION

Not applicable.

## CODE AVAILABILITY

Not applicable.

## Data Availability

Not applicable.
